# Condition-Specific Competitive Effects of the Invasive Mosquito *Aedes albopictus* on the Resident *Culex pipiens* among Different Urban Container Habitats May Explain Their Coexistence in the Field

**DOI:** 10.3390/insects12110993

**Published:** 2021-11-04

**Authors:** Paul T. Leisnham, Shannon L. LaDeau, Megan E. M. Saunders, Oswaldo C. Villena

**Affiliations:** 1 Department of Environmental Science & Technology, University of Maryland, College Park, MD 20742, USA; ms1umd@gmail.com (M.E.M.S.); oswaldo.villena@gmail.com (O.C.V.); 2 Cary Institute of Ecosystem Studies, 2801 Sharon Turnpike, P.O. Box AB, Millbrook, NY 12545, USA; ladeaus@caryinstitute.org

**Keywords:** biological invasion, interspecific competition, mosquitoes, trash, urban greenspace, West Nile virus

## Abstract

**Simple Summary:**

It is important to understand the social and ecological factors that affect mosquito invasions to better assess impacts on resident communities, identify disease risks, and coordinate control efforts. Condition-specific competition, when environmental conditions alter the outcome of competition, can foster the persistence of resident species after the invasion of a competitively superior invader. We test whether condition-specific competition can help the resident *Culex pipiens* persist with the competitively superior invasive mosquito *Aedes albopictus* in water from different urban container habitats. We tested the effects of *A. albopictus* on *C. pipiens’* survival and development in water collected from common functional and discarded containers in Baltimore, Maryland, USA. We found increased densities of *A. albopictus* negatively affected the survivorship and development of *C. pipiens* in water from discarded, but not functional, containers, driven mainly by water from trash cans. These results suggest that the contents of different urban containers alter the effects of *A. albopictus* on *C. pipiens* and that trash cans, in particular, facilitate the persistence of *C. pipiens.* Because *C. pipiens* is the main mosquito species that spreads West Nile virus in many urban areas, controlling its production from trash cans might help manage West Nile virus risks.

**Abstract:**

Condition-specific competition, when environmental conditions alter the outcome of competition, can foster the persistence of resident species after the invasion of a competitively superior invader. We test whether condition-specific competition can facilitate the areawide persistence of the resident and principal West Nile virus vector mosquito *Culex pipiens* with the competitively superior invasive *Aedes albopictus* in water from different urban container habitats. (2) Methods: We tested the effects of manipulated numbers of *A. albopictus* on *C. pipiens’* survival and development in water collected from common functional and discarded containers in Baltimore, MD, USA. The experiment was conducted with typical numbers of larvae found in field surveys of *C. pipiens* and *A. albopictus* and container water quality. (3) Results: We found increased densities of *A. albopictus* negatively affected the survivorship and development of *C. pipiens* in water from discarded containers but had little effect in water from functional containers. This finding was driven by water from trash cans, which allowed consistently higher *C. pipiens’* survival and development and had greater mean ammonia and nitrate concentrations that can promote microbial food than other container types. (4) Conclusions: These results suggest that the contents of different urban containers alter the effects of invasive *A. albopictus* competition on resident *C. pipiens,* that trash cans, in particular, facilitate the persistence of *C. pipiens,* and that there could be implications for West Nile virus risk as a result.

## 1. Introduction

Interspecific competition is often strongly asymmetrical, thereby leading to the exclusion of the weaker competitor [1,2,3], but species can avoid being excluded via several ecological mechanisms, including spatial segregation [4], differential resource use [1], or tradeoffs between competitive ability and environmental tolerance [5,6]. Among the most interesting and widespread mechanisms that promote species coexistence is condition-specific competition, when environmental conditions alter the outcome of competition in favor of a species that is usually a weaker competitor and would otherwise be displaced [1,5,7]. Although most studies of condition-specific competition have focused on the effects of abiotic conditions (e.g., temperature, humidity, chemicals), variation in biotic conditions are also likely to have critical impacts on the outcome of interspecific competition [8].

Biological invasions offer an excellent opportunity to study interspecific competition. They often create nonequilibrium systems in which interspecific interactions are much stronger than in undisturbed systems [9]. Understanding the ecological mechanisms altering competition between invasive and resident species is also of practical importance, especially when they foster the spread and coexistence of an inferior competitor into an introduced range or the persistence of a weaker resident species after the invasion of a competitively superior invader. Among the most tractable and well-studied biological invasions involve urban mosquitoes that utilize water-filled artificial containers at their competing larval life-stage [10,11]. Allochthonous detritus provides the main resource base in container habitats, and strong interspecific competition for nutrients and associated microorganisms typically structures their mosquito communities [8,12]. Container habitats are patchily distributed across urban landscapes, easily sampled for mosquitoes, and species interactions hypothesized to underlie observed trends from the field can be studied in manipulative experiments that yield testable predictions. Many urban mosquitoes are also important vectors of human diseases, and the regional coexistence of species might alter transmission risks, including extending transmission seasons and facilitating pathogen spillover into other host species [12].

The tiger mosquito, *Aedes albopictus* (Skuse) is native to East Asia but has invaded over 30 countries since the 1980s to become the most abundant urban mosquito in many cities around the world [13,14,15,16]. The spread of *A. albopictus* has been attributed to its utilization of a range of containers and its competitive superiority over almost all other species, particularly in habitats with limited resources [12]. Its competition with *Aedes aegypti* (L.) has been well-documented, particular in the southeastern United States [17]. In the northeastern United States, *A. albopictus* regionally coexists with the resident northern house mosquito *Culex pipiens* (L.), another introduced mosquito that first arrived in North America 400 years ago and is now considered a resident species [18,19,20]. Two laboratory studies have tested competition between North American strains of *A. albopictus* and *C. pipiens* and convincingly demonstrate the overwhelming competitive superiority of *A. albopictus* under almost all conditions [21,22], which is consistent with studies in Europe [13,23], but see also [24].

Despite such apparent competitive superiority, *C. pipiens* has managed to persist in many urban areas after *A. albopictus* invasion [18,21,25]. One mechanism facilitating *C. pipiens* coexistence with *A. albopictus* may be habitat segregation. *C. pipiens* can be collected from a wider variety of habitats than *A. albopictus*, including ground pools and subterranean habitats, where *A. albopictus* is collected in much lower densities, and thus may escape sufficient competition from the invader to allow it to persist [23]. This potential for habitat separation is likely one reason why the competitive effects on *C. pipiens* from *A. albopictus* has received relatively little attention compared to other resident mosquitoes that solely utilize the same containers as *A. albopictus* (e.g., *A. aegypti)*. However, in many urban areas, above-ground containers appear to be the only available larval habitats for *C. pipiens*, and it frequently co-occurs in the same individual containers with *A. albopictus,* where it is likely to experience strong interspecific competition [17,25]. Competition experiments between North American *A. albopictus* and *C. pipiens*, as well as those between other strains of the species, have used artificial resource levels and densities likely to elicit strong competition in order to demonstrate which species has a competitive advantage [21,22,23]. No experiments have tested competition between *A. albopictus* and *C. pipiens* at densities and habitat conditions found in the field. The terrestrial environment surrounding urban container habitats can vary considerably by vegetation, shade, and temperature, among other things, which can all alter water conditions. Moreover, the type of container can further reflect its interaction with the environment. For example, discarded containers, including dumped tires, are often discarded in shady locations under dense vegetation and are rarely disturbed, whereas functional containers, including buckets for gardening, are often regularly emptied and stored in tidied areas close to buildings. This heterogeneity in container type might support condition-specific competition to result in local variation in the success and impact of invasive species [21,26,27] or facilitate species coexistence of weaker competitors [7,28].

This study tests two hypotheses central to understanding the persistence of resident *C. pipiens* with invasive *A. albopictus* in a diverse urban landscape. First, we test the hypothesis that interspecific competition is important in conditions typical to those *A. albopictus* and *C. pipiens* commonly experience in urban containers. Second, we test the hypothesis that variation in nutrient conditions among specific container types can alter the outcome of competition, and that this may facilitate the areawide persistence of *C. pipiens* with *A. albopictus.* These hypotheses generate three predictions that we tested in field surveys in Baltimore, Maryland, USA, and a controlled competition trial in the laboratory. We predict that, in the field, *A. albopictus* and *C. pipiens* co-occur in containers and that the proportion of larvae that are *C. pipiens* varies with container type. In the competition trial, we predict strong competition among larvae at densities per container that are observed in the field, and that competition varies among nutrient conditions found in different container types.

Understanding the conditions that favor the coexistence of *A. albopictus* and *C. pipiens* in urban ecosystems is of significant public health importance. *C. pipiens* is the principal vector of West Nile virus (WNV) in the northeastern United States, circulating and amplifying the virus among local bird populations [29] and for their significant role in bridging WNV and other arboviruses into human populations [30,31]. The persistence of *C. pipiens* after the invasion of *A. albopictus* in urban areas where WNV is present is likely to maintain existing enzoonotic circulation and human transmission of the virus. Although *A. albopictus* is much less efficient at amplifying WNV than *C. pipiens,* it is a more aggressive human biter and is likely an additional bridge of the virus from bird to human populations [32,33,34,35,36]. Therefore, the coexistence of *C. pipiens* with *A. albopictus* in urban environments is expected to increase local WNV transmission risk and help us better understand ecological mechanisms facilitating such coexistence of epidemiological importance.

## 2. Materials and Methods

### 2.1. Field Surveys

We systematically searched and described all accessible water-filled containers on 33 city blocks in West Baltimore, MD, USA in 2015 and identified the most frequently observed container types with juvenile (larvae, pupae) mosquitoes [17]. For this study, we focused on six container types: three types of unmanaged discarded containers found on parcels with vacant land (i.e., no building) or abandoned buildings, and three types of functional containers found in yards of resident-occupied parcels (Table 1). These were the three most common water-filled functional (fence pole, trash can, bucket) containers, constituting 57.3% (1504/2624) of total containers. During three seasonal periods (early season, May; middle season, July–August; late season, September) in 2015, we sampled all accessible containers in 12 city blocks. For each container, we homogenized its contents (water and detritus) and extracted a representative sample up to 1.0 L. Collected mosquito larvae were brought back to the laboratory, preserved in ethanol, enumerated, and identified by development stage. We identified a representative sample of up to 50 third and fourth instar mosquito larvae to species, and up to 50 first and second instar larvae to genus, using an established key [36]. Species abundances of first and second instars were estimated based on relative species abundances of co-occurring third and fourth instar larvae within the same genus (Dataset S1). The mean density of larvae per occupied container across the six focal container types over the entire season was 0.267 larva per mL (Table 1), and we used this field density to calculate our baseline number of added larvae for the laboratory experiment (see below).

### 2.2. Competition Trial

The main goal of our experiment was to manipulate numbers of *A. albopictus* larvae per individual container and to determine the effects of these manipulations on survivorship and development of *C. pipiens* in aquatic conditions from each of the six container types. The water used in the competition trial was collected from a representative four individual containers from each of the six focal container types on 16 and 17 September 2016. We sampled the first four accessible containers that we encountered from eight randomly selected parcels that were vacant (discarded containers) or occupied (functional containers). Only one container was sampled from any individual parcel and containers were sampled irrespective of observed mosquitoes or any other environmental variables, with the exception of having a minimum 0.5 L water. Prior observations suggested that containers need to hold at least 0.5 L of water to support mosquito development from egg hatching to adulthood without evaporating dry under most conditions. Therefore, we restricted our sampling to containers from which we could extract a representative sample of between 0.5–1.0 L, following the same procedures as in 2015. The contents from each container were stored in a separate sample container (Nalgene, Rochester, NY, USA) and brought back to the laboratory for immediate processing. For most containers, including all discarded plastic, discarded styrofoam, and most buckets, we were able to empty their contents directly into the sample container. For containers that could not be emptied, including all dumped tires, trash cans, and some buckets, we homogenized their contents (water and detritus) and took a 1 L sample. In fence poles, we observed very little organic material but did see rusted metal flakes in the water column, which were easily extracted with water using a turkey baster.

**Figure 1:**
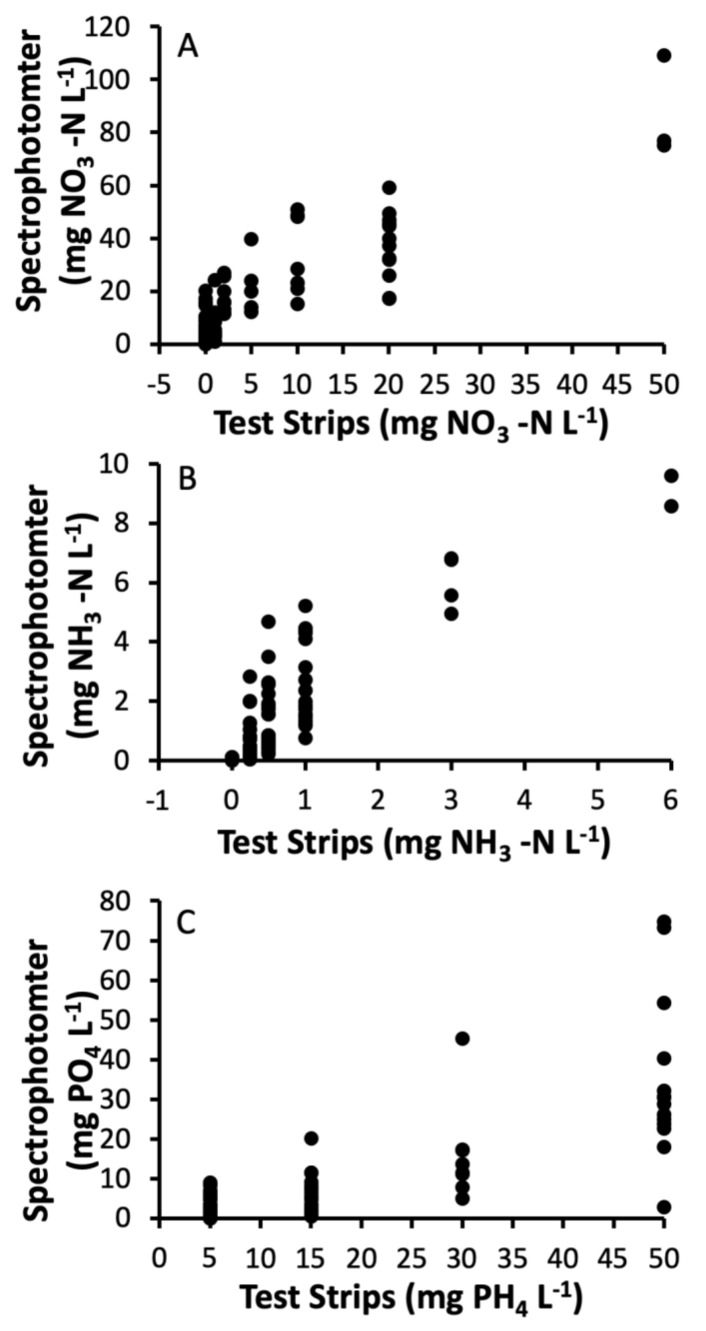
Spectrophotometer versus chemical test strip estimates of dissolved (**A**) nitrate, (**B**) ammonia, and (**C**) phosphate in water samples from water-holding containers.

Within 24 h of collection, all samples (24 total) were sieved (106 µm) to remove any coarse material and larvae. Samples were left standing for another 24 h before being re-checked for larvae a second time. Water nitrate, ammonia, and phosphate concentrations were measured from each sample immediately upon returning to the laboratory and before being sieved using AquaChek Test Strips (Hach Company, Loveland, CO, USA) (Dataset S2). AquaChek Test Strips have been used in past studies to discriminate across broad differences in nutrients among aquatic habitats, e.g., [25,37]. However, we are aware of only one independent study to test the reliability of test strips against laboratory-based standard methods [38]. Another study, Dowling et al. [25], suggested strong correlations with the water quality results of container habitats with appropriate test kits on a spectrophotometer, but the data was not reported and was based on a relatively small number of samples (30–40) (P.T. Leisnham, personal observation). Therefore, in this paper, we use data collected in a prior study to more rigorously test the relationship of AquaChek Test Strips with respective tests on a Hach DR3800 spectrophotometer (Hach, Loveland, CO, USA). In the summer of 2011, water samples were collected from 100 artificial container habitats in residential yards in Centreville, MD, USA. Nitrate, ammonia, and orthophosphate (hereafter phosphate) were tested both using nitrate, ammonia, and phosphate AquaChek Test Strips and a Hach DR3800 spectrophotometer with appropriate low to high reading test kits (Hach TNT830-836). All tests using test strips were carried out within 12 h of collection (Dataset S2). Paired tests on the spectrophotometer were done within 1 week, after a 100 mL subsample had been acidified at pH 2.0 and refrigerated within a few hours of collection. Mean estimates for all three water quality parameters differed between the test strips and the spectrophotometer, with the test strips recording lower values for nitrate and ammonia and higher values for phosphate (Table 2). Nevertheless, estimates for all three parameters were highly correlated (Table 2 and Figure 1), suggesting that the test strips are acceptable for detecting relative nutrient concentrations and available food resources among mosquito container habitats.

After we measured water quality and sieved contents, each of the four replicate samples from the six study container types (24 total) were divided into three 100 mL cups consisting of 90 mL sample water to yield 72 total experimental units. Based on field densities of occupied containers (mean: 0.267 larvae per mL), we calculated the baseline number of larvae in our experiment at 30 larvae per cup at a density of 0.333 larvae per mL. Observed mean densities of larvae in field containers are likely to be an underestimate of the densities of larvae at hatching and represent only the survivors of larger cohorts of hatching larvae. Estimating larval mortality in the field is difficult but it can be high, and *C. pipiens’* mortality can approach 100% under severe resource limitation [39,40]. Hence, our baseline density that is 24.7% higher than the observed mean density in the field is likely a conservative estimate of the density at hatching. For all cups we added newly hatched (<24 h old) larvae of *C. pipiens* at 1/2 the baseline number (i.e., 15). Treatments were defined by the number of newly hatched *A. albopictus* that we added. The three cups from each of the 24 container samples received one of three treatments. The “low” density treatment received no *A. albopictus* (i.e., it had only *C. pipiens* at half the baseline number). The “control” treatment received *A. albopictus* at half the baseline number (i.e., 15), so that total number of mosquito larvae was the baseline number (i.e., 30). The “high” density treatment received *A. albopictus* at the full baseline number, so that total mosquito larvae number was equal to 1.5× the baseline number (i.e., 45). Each density treatment was replicated four times for each of the six container types for a total of 72 experimental units (Dataset S3).

Container habitats may be regularly provisioned with detrital resources in the field, and this provisioning may vary among container types. Because our goal was to compare water conditions found in the field, and because we could not simulate the range of detrital additions that may occur across container types, we chose to not reprovision any treatment but to end the experiment after 6 days. Competition among individuals is strong during early larval development, and six days is often sufficient for maturation to adulthood under ideal conditions [41]; thus, we think our study focused on the period of time when container contents are most likely to regulate resource competition between *A. albopictus* and *C. pipiens*.

### 2.3. Statistical Analyses

Associations between *A. albopictus* and *C. pipiens* in field containers during each of the early, middle, and late seasonal periods, as well as throughout the entire season, were tested using Mantel–Haenszel tests on multiple 2 × 2 tables for each of the six container types [41]. Because *C. pipiens* is the focal species and *A. albopictus* is the associate species, statistical analyses of the competition trial focused only on the response of *C. pipiens* in different container source water to the manipulation of *A. albopictus* abundance [42]. Thus, *A. albopictus* is present only as a treatment. To assess both the survival of *C. pipiens* and its development, we analyzed the proportion of *C. pipiens* surviving to the end of the experiment (arcsine transformed, to meet assumptions of normality and homogeneity of variances) and mean developmental stage (instar = 1, 2, 3, 4, pupa = 5), by ANOVA. Container types selected for this study were not a random sample of all possible container types. Therefore, container type was treated as a fixed effect with treatment and interaction, and statistical inferences extend only to the container types selected. Although we thoroughly searched all experimental cups for field larvae over two days, there were a few cases of incomplete removal. We chose to omit from analyses 3 cups in which *A. albopictus* recovered at the end of the experiment were more than the stocked number by >3 individuals, resulting in 69 useable cups for analyses. There were no cases where recovered *C. pipiens* exceeded experimentally added numbers. MANOVA was used to test differences in nitrate, ammonia, and phosphate concentrations (all log10 transformed to meet assumptions of normality and homogeneity of variances) among container types that were sampled for water used in the competition trial. We used F statistics derived from Pillai’s Trace to detect differences and interpreted contributions of dependent variables to significant MANCOVA effects using standardized canonical coefficients (SCCs) [43]. For all ANOVAs and the MANOVA, we tested for significant differences among container types using pairwise contrasts [43] with sequential Bonferroni correction for all possible comparisons within each analysis. We used a priori contrasts to compare mean values of *C. pipiens’* survivorship and instar, and water quality (nitrate, ammonia, and phosphate concentrations) between functional vs. discarded container types.

## 3. Results

### 3.1. Field Surveys

As predicted, *A. albopictus* and *C. pipiens* commonly co-occurred in individual containers but were most frequently associated in two discarded container types, discarded plastic and dumped tires, and two functional types, buckets and trash cans, where they also increased in the proportion of habitats that they were found together from early to late season (Table 1). *Aedes albopictus* and *C. pipiens* were not associated with each other in either the early (χ^2^-values = 0.071–2.177, *p*-values = 0.1401–0.7890) or middle (χ^2^-values = 0.040–0.333, *p*-values = 0.5640−0.8410) season but were commonly collected together in functional containers in the late season (χ^2^ = 7.369, *P* = 0.0066; discarded containers: χ^2^ = 1.012, *p* = 0.3140). *C. pipiens* rarely occurred with *A. albopictus* in fence poles (4/63) and discarded styrofoam (1/19), nor was it frequently observed in these habitats on its own (fence poles: 3/63; discarded styrofoam: 2/19), suggesting that these were generally unfavorable habitats for the resident species. *C. pipiens* also declined as a proportion of total collected larvae from early to late season, while the competitively superior *A. albopictus* increased in proportion during this same time (Table 1). However, the seasonal decline of *C. pipiens* was milder in the functional container types, trash cans (0.907 to 0.455; 49.8% decline) and buckets (0.692 to 0.257; 63.9% decline), than in discarded plastic (0.957 to 0.257; 73.1% decline) and dumped tires (0.921 to 0.255; 72.3% decline).

### 3.2. Competition Trial

**Figure 2:**
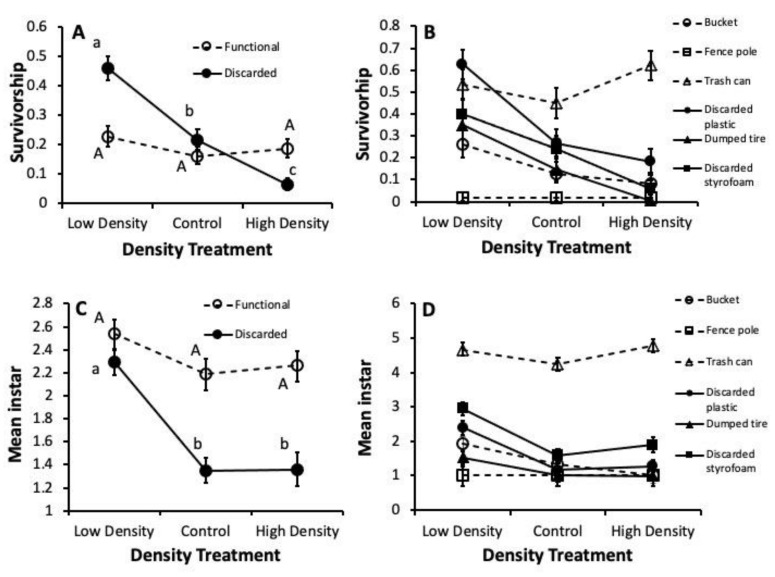
Least Squares Means (+SE) for competition treatments across broad container categories and container types for (**A**,**B**) survival of *C. pipiens* and (**C**,**D**) mean of instar of surviving *C pipiens*. Means within container categories associated with the same letter are not significantly different (Bonferroni test, experimentwise α = 0.05). Different means within container types are not denoted with letters for clarity.

There were significant Container Type x Density interaction effects on larval survival (F_10,51_ = 3.43, *p* = 0.0017) and development (F_10,41_ = 2.80, *p* = 0.0097), indicating that the effects of *A. albopictus* densities on *C. pipiens* performance depended on the water conditions in different container types. *C. pipiens’* survival increased with reduced *A. albopictus* density in discarded container water but was not responsive to *A. albopictus* density in water from functional containers (Figure 2A). Survival declined from low- to high-density treatments in water from discarded containers (Figure 2B). The low-density treatment differed from both the control (t_51_ = −3.09, *p* = 0.0032) and high-density (t_51_= −4.02, *p* = 0.0002) treatments in water from discarded plastic, and the low-density treatment differed from high-density treatments in water from dumped tires (t_51_ = −4.86, *p* < 0.0001) and in water from discarded styrofoam (t_51_ = −4.34, *p* < 0.0001). Mean survival across all treatments was highest in water from trash cans relative to all other individual container types (t_51_-values = −11.91–3.37, *p*-values < 0.0001). In contrast, survival was significantly lower for larvae in water from fence poles than in all other container types (t_51_-values = −11.91–4.15, *p*-values < 0.0001–0.0019). Survival did not vary with density treatment in any of the three functional container types (*p*-values > 0.5000).

For larvae development, the pattern of differences among treatments was broadly similar to that of survival (Figure 2C). Instars were smaller in the high-density and control treatments compared to the low-density treatments in water from discarded containers but were alike across density treatments in water from functional containers. Mean treatment differences in water from discarded containers were mainly driven by differences in discarded plastic and styrofoam, with significantly smaller instars in the high-density treatment than the low-density treatment in water from discarded plastic (t_41_ = −4.10, *p* = 0.0002) and in the high-density treatment than the control in water from discarded styrofoam (t_41_ = −4.99, *p* < 0.0001). Similar to survival, mean development (and resultant body size) across all densities was clearly highest in water collected from trash cans (Figure 2D), being significantly greater than in water from all other individual container types (t_41_-values = −18.47–14.94, *p*-values < 0.0001)

### 3.3. Nutrient Analyses

**Figure 3:**
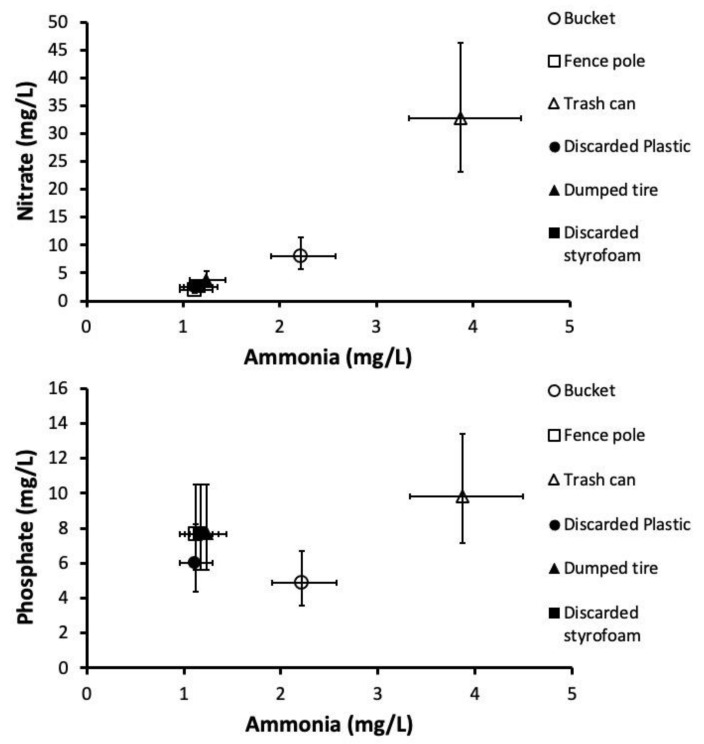
Bivariate plot of container water quality.

Ammonia (*r* = 0.27; 0.2004) and nitrate (*r* = 0.36; 0.0818) concentrations were not associated with phosphate concentration but were highly associated each other (*r* = 0.82; *p* < 0.0001) within individual containers. MANOVA on water quality showed a significant effect of container type, which most strongly affected ammonia, moderately affected nitrate, and only weakly affected phosphate concentrations (Table 3). Multivariate pairwise contrasts showed overall mean water quality differences between functional vs. discarded container types, due mostly to differences in ammonia concentration and, to a lesser extent, nitrate concentration (Table 3). Trash cans (*p*-values < 0.0001–0.0003) and buckets (*p*-values = 0.004–0.0120) appeared to drive this difference, with pairwise contrasts revealing them as different from all other container types (but not themselves; *p* = 0.0665). Trash cans had clearly higher ammonia (SCCs: 1.23–1.53) and moderately higher nitrate (SCCs: 0.76–0.96) concentrations, whereas buckets had higher ammonia (SCCs: 1.14–1.77) and nitrate (SCC: 0.36–0.84) concentrations but also marginally lower phosphate concentrations compared to some other container types (SCCs: −0.65–0.76) (Figure 3).

## 4. Discussion

Our results demonstrate clear evidence of condition-specific competition through the moderating effects of heterogeneous container habitat across an urban landscape that alters the outcome of *A. albopictus* competition on resident *C. pipiens.* In a laboratory competition trial, survival and development of *C. pipiens* was most affected by increased densities of *A. albopictus* in water sourced from discarded containers but had little effect in water sourced from functional containers. The numbers of larvae per experimental cup were determined by observed numbers in occupied field containers, indicating that such variation in interspecific competition is important under typical field conditions. The lack of *A. albopictus’* competitive effects on *C. pipiens* in water from functional containers was mainly driven by the results from trash cans, which allowed consistently higher *C. pipiens* survival and development than in other container types that did not vary with *A. albopictus’* density. The effect on survival is particularly important as survival is the life history variable most closely associated with fitness and most directly related to the potential for local extinction of a species [44]. Our study here is the first to have tested competition between *A. albopictus* and *C. pipiens* in aquatic conditions and at larval densities found in urban environments where these species typically coexist and offers condition-specific competition as a plausible mechanism for the persistence of *C. pipiens* despite the invasion of the competitively *A. albopictus.*

The capacity for *C. pipiens* to maintain survival and growth in the presence of increasing densities of *A. albopictus* may be associated with the nutrient concentrations in specific container types. Mean ammonia and nitrate concentrations were greater in water from functional containers, particularly from trash cans where concentrations were over two times those in discarded plastic, dumped tires, and discarded styrofoam. The competitive superiority of *A. albopictus* is likely a driver in the observed decline of *C. pipiens’* abundances from early to middle and late season in the field. However, this decline appeared to be milder in two of the three functional container types (trash cans, buckets) compared to two of the three discarded container types (discarded plastic, dumped tires), in which both species commonly cooccurred. Overall, these results support our hypotheses that interspecific competition between *A. albopictus* and *C. pipiens* is important in conditions typical to what these species interact in urban containers and that container type moderates the competitive outcome. All other experiments of competition between *A. albopictus* and *C. pipiens* have involved highly manipulated resource levels [13,21,22,23,24]. Those focused on the North American strains of these species have shown the clear competitive superiority of *A. albopictus* over *C. pipiens,* even at treatments representing very high resource levels, including fresh grass clippings [22]. This study did not measure microbial communities (bacteria, fungi) that serve as the trophic link between nutrients and mosquito larvae in container habitats. Microbial measurements require the collection of water samples, which would have been difficult in this study, given the need to use the entire water contents of containers for the competition trial and the small size of many habitats. However, concentrations of nutrients, particularly nitrate and ammonia, have been shown to strongly predict microbial productivity, and likely provide a robust overall comparison of available food resources for mosquito larvae across containers [8,12,45]. Nevertheless, future studies should consider characterizing microbial communities from urban containers to better understand how overall microbial productivity and diversity may vary with nutrients to affect interspecific mosquito competition among container types.

Our experiment, as in all other experiments testing competition between these species, excluded the effects of other aquatic invertebrates that may alter competition. Studies on the competitive interactions between *A. albopictus* and other container mosquitoes (e.g., *A. aegypti, Aedes triseriatus* (Say)*, Aedes japonicus* (Theobald) have shown that predators, including *Toxorhynchites rutilus* and *Corethrella appendiculata* [46,47,48,49] and parasitic protozoa in the genus *Ascogregarina* [50] can alter their competitive interactions. Some authors have also suggested that the larvae of a third mosquito species or other detritivores may alter the competitive effects of *A. albopictus* [12]. However, although the effects of other species may be important in other systems, they are unlikely to play a substantive role in modifying competition between *A. albopictus* and *C. pipiens* in urban environments. These species cooccur in urban areas at temperate latitudes where container invertebrate diversity is particularly low. Numerous studies in Baltimore, e.g., [51,52] and other cities in the northeastern United States, e.g., [25,53] and parts of Europe, e.g., [23,54], show that these species constitute the vast majority of total invertebrate larvae in urban containers. This finding and the fact that alterative habitats (e.g., ground pools, subterranean habitats) are often substantially fewer than above ground water-filled containers in Baltimore [51], and other cities [55], further strengthens the case of condition-specific competition among container types as a primary mechanism for the persistence of *C. pipiens* after *A. albopictus* invasion in urban areas.

While most abundant in trash can containers, *C. pipiens* were not observed in either fence poles or discarded styrofoam containers during the 2015 survey, suggesting that these habitats are unlikely to play a role facilitating the coexistence of *C. pipiens* with *A. albopictus*. These two container types had lower nutrient concentrations than trash cans but similar concentrations than in discarded plastic and dumped tires, which yielded higher densities of both mosquito species, suggesting that nutrients were not a limiting factor. Fence poles and styrofoam containers could have elevated toxins (e.g., rust from poles or microplastics from discarded styrofoam) that negatively affect *C. pipiens’* survival, but this is unlikely because *C. pipiens* is among the most tolerant urban mosquito species to a wide range of water conditions [56,57] and *A. albopictus* is generally considered less tolerant to environmental toxins [58] and was collected from these container types. A more likely reason is that fence poles and discarded styrofoam are among the smaller container types sampled, and *A. albopictus* more readily oviposits in small habitats. The diameter of all fence poles was 5 cm, and discarded styrofoam was usually of similar size, considerably below the size that *C. pipiens* typically oviposits in but on that is commonly utilized by *A. albopictus* [23]. Interestingly, we found little evidence of an association (negative or positive) between *A. albopictus* and *C. pipiens* in discarded field containers and in early and middle season sampling periods. Interspecific competition between *C. pipiens* and *A. albopictus* in container habitats appears most important later in the season (July onwards) when the two species were more commonly found cooccurring in the same individual containers. This result could be because of several reasons. The lack of association in the early season is probably due to low area-wide *A. albopictus* abundances because of later overwintering emergence [59,60]. The lack of association in the middle season, when *A. albopictus* made up a greater proportion of total larvae, and in discarded containers, was likely either the result of habitat segregation due to different oviposition preferences or the result of interspecific competition [23].

The ecological findings of this study likely have important implications for public health and the management of *C. pipiens*. *Culex pipiens’* success in escaping the competitive effects of *A. albopictus* in functional containers suggest that these container types could be targeted by control efforts to reduce WNV risk in many urban areas. *C. pipiens* is the principal vector for WNV in urban areas in the northern United States and Europe, circulating and amplifying the virus among bird populations [29,31,61] and bridging WNV into human populations [30]. The displacement of *C. pipiens* in urban areas by *A. albopictus* may be expected to reduce WNV transmission, although *A. albopictus* can serve as a vector for other arboviruses. The role of *A. albopictus* in the transmission of several arboviruses in the United States is still unclear, but several arboviruses have been isolated from field individuals, including eastern equine encephalitis, La Crosse, chikungunya, and Zika viruses [14,62,63]. On the other hand, the persistence of *C. pipiens* after *A. albopictus* invasion is likely to increase WNV risk because *A. albopictus* probably plays an additional role in bridging WNV into human populations. Trash cans (including recycling bins) used by most homes may be particularly important habitats in WNV amplification and human transmission if they facilitate *C. pipiens’* persistence. In this study, the nutrients that accumulate in trash cans may have helped to relax competitive effects from *A. albopictus* and provide favorable developmental conditions to produce large abundances of biting adults. Water collected from trash cans for the competition trial in this study had significantly higher nutrients than almost all other container types (except buckets), presumably because of remnant organic content even after emptying, which was evident during field collections (P.T. Leisnham, personal observation). Furthermore, their plastic material and frequent location in open sunlight are likely to result in higher temperature water in the field, and combined with their large volume, probably strengthen their contribution to the areawide production of *C. pipiens*. Although lidded, we observed most trash cans left open after being emptied by municipal waste management. Many trash cans have drainage holes, but we frequently observed these blocked by residual organic content, allowing them to hold water after recent rainfall. The combination of these environmental conditions and human behaviors likely provides an excellent habitat for *C. pipiens.*

## 5. Conclusions

This study is the first to have tested competition between *A. albopictus* and *C. pipiens* in aquatic conditions and at larval densities found in urban environments where these species typically coexist. It shows that the contents of different urban containers alter the effects of *A. albopictus* on *C. pipiens* and that trash cans, in particular, facilitate the persistence of *C. pipiens*, despite the invasion of the competitively *A. albopictus.* It offers condition-specific competition as a plausible mechanism for the persistence of *C. pipiens,* which may also have implications for West Nile virus transmission and risk.

## Figures and Tables

**Table 1:** Sampled density of mosquito larvae per container in each container type in 2014. Mean densities per container and SD are determined for occupied and sampled containers only.

								Observed Density Per Container	
Container Type	Total Number of Water-Filled Containers	Sampled Containers	*Aedes albopictus*	*Culex pipiens*	*Aedes albopictus* + *Culex pipiens*	Neither	Proportion *Culex pipiens* (N)	Mean Density (Larvae/mL)	SD
Early season (May)									
Bucket	38	5	2	3	0	0	0.692 (45)	0.065	0.045
Fence pole	78	11	2	2	3	4	0.813 (87)	0.149	0.286
Trash can	90	14	1	11	1	1	0.907 (567)	0.284	0.218
Discarded plastic	203	32	1	18	4	9	0.957 (667)	0.161	0.175
Dumped tire	75	46	2	27	7	10	0.921 (1136)	0.242	0.375
Discarded styrofoam	77	7	0	2	0	5	0.936 (117)	0.558	0.323
Middle season (July–August)									
Bucket	41	19	7	3	5	4	0.621 (174)	0.154	0.163
Fence pole	85	25	15	0	1	9	0.013 (147)	0.295	0.491
Trash can	56	18	6	5	7	0	0.556 (266)	0.197	0.258
Discarded plastic	112	46	21	4	17	4	0.332 (449)	0.366	0.554
Dumped tire	72	59	29	3	22	5	0.162 (308)	0.316	0.359
Discarded styrofoam	50	12	10	0	1	1	0.116 (41)	0.886	0.816
Late season (September)									
Bucket	46	11	4	2	5	0	0.257 (38)	0.217	0.181
Fence pole	129	27	26	1	0	0	0.005 (1)	0.154	0.198
Trash can	71	15	5	4	6	0	0.455 (80)	0.163	0.183
Discarded plastic	99	11	6	0	4	1	0.276 (214)	0.234	0.151
Dumped tire	57	27	13	4	10	0	0.255 (130)	0.228	0.232
Discarded styrofoam	31	0	-	-	-	-	-	-	-

**Table 2:** Mean (SD) nitrate, ammonia, and phosphate concentrations of water-filled urban containers as measured by the two methods, AquaChek Test Strips and a Hach DR3800 spectrophotometer. Differences and correlations between values from the two methods were tested using paired *t*-tests and Pearson correlations, respectively.

Nutrient	Test Strip (mg/L)		Spectrophotometer (mg/L)		df	t	*P*	*r*	*p*
	Mean	SD	Mean	SD					
Nitrate	5.21	10.34	15.64	19.21	98	−9.30	<0.0001	0.885	<0.0001
Ammonia	0.67	0.99	1.52	1.95	94	−6.82	<0.0001	0.855	<0.0001
Phosphate	15.96	16.09	8.80	14.44	93	6.89	<0.0001	0.787	<0.0001

**Table 3:** MANCOVA results and standardized canonical coefficients for container water quality.

				Canonical Variates			Standardized Canonical Coefficients		
Source of Variation	Pillai’s Trace (F)	df	*p*	Variate Number	Percent Variation	*p*	Nitrate	Ammonia	Phosphate
Container type	1.87	15, 54	0.0481	1	95.6	0.0017	0.89	1.42	−0.53
				2	3.4	0.8680	0.72	−0.76	0.88
				3	1.0	0.8314	−1.71	−1.62	0.41
Functional vs. Discarded	9.86	3, 16	0.0006	1	100	0.0006	0.69	1.60	−0.54

## Data Availability

The data presented in this study are available in Dataset S1: Field survey, Dataset S2: Nutrient analyses, Dataset S3: Competition trial.

## Supplementary Materials

The following are available online at https://www.mdpi.com/article/10.3390/insects12110993/s1, Dataset S1: Field survey, Dataset S2: Nutrient analyses, Dataset S3: Competition trial.
